# Advances in Cardiac Pacing: Arrhythmia Prediction, Prevention and Control Strategies

**DOI:** 10.3389/fphys.2021.783241

**Published:** 2021-12-02

**Authors:** Mehrie Harshad Patel, Shrikanth Sampath, Anoushka Kapoor, Devanshi Narendra Damani, Nikitha Chellapuram, Apurva Bhavana Challa, Manmeet Pal Kaur, Richard D. Walton, Stavros Stavrakis, Shivaram P. Arunachalam, Kanchan Kulkarni

**Affiliations:** ^1^Department of Cardiovascular Diseases, Mayo Clinic, Rochester, MN, United States; ^2^Division of Gastroenterology and Hepatology, Mayo Clinic, Rochester, MN, United States; ^3^Department of Medicine, GAIL, Mayo Clinic, Rochester, MN, United States; ^4^IHU LIRYC, Electrophysiology and Heart Modeling Institute, Fondation Bordeaux Université, Bordeaux, France; ^5^Centre de Recherche Cardio-Thoracique de Bordeaux, University of Bordeaux, Bordeaux, France; ^6^INSERM, Centre de Recherche Cardio-Thoracique de Bordeaux, Bordeaux, France; ^7^Heart Rhythm Institute, University of Oklahoma Health Sciences Center, Oklahoma City, OK, United States; ^8^Department of Radiology, Mayo Clinic, Rochester, MN, United States

**Keywords:** arrhythmias, pacing, alternans, control, non-linear dynamics, prediction

## Abstract

Cardiac arrhythmias constitute a tremendous burden on healthcare and are the leading cause of mortality worldwide. An alarming number of people have been reported to manifest sudden cardiac death as the first symptom of cardiac arrhythmias, accounting for about 20% of all deaths annually. Furthermore, patients prone to atrial tachyarrhythmias such as atrial flutter and fibrillation often have associated comorbidities including hypertension, ischemic heart disease, valvular cardiomyopathy and increased risk of stroke. Technological advances in electrical stimulation and sensing modalities have led to the proliferation of medical devices including pacemakers and implantable defibrillators, aiming to restore normal cardiac rhythm. However, given the complex spatiotemporal dynamics and non-linearity of the human heart, predicting the onset of arrhythmias and preventing the transition from steady state to unstable rhythms has been an extremely challenging task. Defibrillatory shocks still remain the primary clinical intervention for lethal ventricular arrhythmias, yet patients with implantable cardioverter defibrillators often suffer from inappropriate shocks due to false positives and reduced quality of life. Here, we aim to present a comprehensive review of the current advances in cardiac arrhythmia prediction, prevention and control strategies. We provide an overview of traditional clinical arrhythmia management methods and describe promising potential pacing techniques for predicting the onset of abnormal rhythms and effectively suppressing cardiac arrhythmias. We also offer a clinical perspective on bridging the gap between basic and clinical science that would aid in the assimilation of promising anti-arrhythmic pacing strategies.

## Introduction

Sudden cardiac death (SCD) instigated by cardiac arrhythmias is one of the leading causes of mortality worldwide, accounting for about 300,000 deaths annually in the United States alone (Srinivasan and Schilling, 2018). Atrial tachyarrhythmias (abnormally faster atrial activation rates) such as atrial flutter and atrial fibrillation (AF), although non-fatal are associated with substantial complications, most commonly thromboembolic (cerebral, extremities, or visceral) leading to increased risk of strokes, limb ischemia, organ infarctions and hence, hospitalizations (Petersen, 1990). Bradyarrhythmias (abnormally slower heart rates) originating due to disorders of the atrial conduction pathways may either lead to morbidity or progress to SCD by instigating ventricular tachycardia (VT) or asystole (Khurshid et al., 2018). Ventricular tachyarrhythmias (VT/VF) on the other hand can be lethal and are the primary cause of SCD, mandating immediate clinical intervention. About 1.5–5% of the general population is currently afflicted by arrhythmias, either atrial or ventricular (Desai and Hajouli, 2021).

The past decade has seen an abundance of medical devices aiming to restore normal cardiac rhythm with new pacing strategies being designed to apply electrical stimulations for the prevention and suppression of arrhythmias. From anti-tachycardia pacing and novel pacemaker algorithms, to cardiac resynchronization therapy and implantable defibrillators, multiple electrical stimulation modalities have been proposed and implemented for arrhythmia management (Trohman et al., 2004). Conventional non-physiological pacing from the right ventricular apex has shown reliable results in the treatment of bradyarrhythmias, however, its adverse hemodynamics of dyssynchronous ventricular contraction predisposes the patients to develop AF, cardiomyopathy, heart failure (HF) or death (Vijayaraman et al., 2017; Arora and Suri, 2021; Perla et al., 2021). An alternative approach is physiological cardiac pacing, which transmits electrical impulses via the normal conduction pathway (Arora and Suri, 2021) and has shown a relative risk reduction of 27% for new onset chronic AF compared to ventricular pacing (Skanes et al., 2001; Trohman et al., 2004), yet technical challenges hinder its application (Arora and Suri, 2021).

Technological advances in pacing have enabled rate responsive or adaptive pacing which successfully tackle chronotropic incompetence wherein the sino-atrial node is unable to increase the heart rate to meet the changing metabolic demands (Trohman et al., 2004, 2020). Most modern devices with adaptive pacing have sensors to detect physiological or physical indices of activity and simulate normal sino-atrial nodal response (Trohman et al., 2004, 2020). Yet, despite the advances in cardiac arrhythmia treatment modalities, given the spatiotemporal and structural complexity of the human heart, designing algorithms to effectively control the heart rate and prevent fatal rhythms has been challenging. In addition, although implantable cardioverter defibrillator (ICD) shocks and anti-tachycardia pacing techniques can protect individuals from life threatening arrhythmias, excessive and inappropriate pacing or shocks have detrimental effect, ranging from impaired myocardial contraction leading to systolic dysfunction, progressive HF, unnecessary drug treatment, proarrhythmogenicity, reduced quality of life and psychological impacts on the patient (Germano et al., 2006; Bhavnani et al., 2010).

Thus, there is a pressing need to better understand cardiac dynamics, the initiation of unstable rhythms in the heart, and subsequently, their prevention to minimize unnecessary shock and drug treatments. Here, we present a systematic review of the state-of-the-art cardiac pacing methodologies aimed at predicting and controlling cardiac arrhythmias. We present a clinical perspective on the current arrhythmia prevention strategies and discuss the efforts to bridge the gap between translational and clinical science that would enable the assimilation of novel pacing modalities.

## Search Strategy

Multiple databases including Google Scholar and PubMed, were comprehensively searched without language and time restriction. For prediction techniques, the literature search was focused on traditionally used clinical methods as well as novel techniques incorporating non-linear dynamical and artificial intelligence (AI) models. The following keywords were used as search criteria: “(clinical markers OR deep learning OR artificial intelligence OR machine learning OR EP study OR non-linear dynamics OR clinical diagnosis OR prediction) AND cardiac arrhythmias.” For arrhythmia prevention and control methods, the literature search was focused on clinical and translational pacing modalities for suppressing abnormal cardiac rhythms. The following keywords were used as search criteria: “(prevention OR control OR suppression OR overdrive pacing OR biventricular pacing OR autonomic control OR stochastic pacing OR diastolic interval pacing OR novel pacing method) AND cardiac arrhythmias”.

## Management of Atrial Arrhythmias

### Prediction Modalities

#### Traditional Clinical Methods

Various clinical tests and devices are traditionally used to diagnose/predict arrhythmias including ambulatory electrocardiogram (ECG) monitors and event recorders, *trans*-telephonic transmitters, stress tests, echocardiography, electrophysiology (EP) studies, or tilt table tests (Clinic, 2018). ECGs remain the predominant clinical tool for monitoring cardiac electrical activity and predicting the onset of cardiac arrhythmias (Castro-Torres et al., 2015).

Holter monitoring is a prominent diagnostic method used to monitor the heart continuously for 24–72 h (Clinic, 2021). This technique was introduced in the 1960’s and has been used since as an integral tool in cardiac arrhythmia prediction (De Asmundis et al., 2014). There are limitations of Holter monitoring if the arrhythmias only occur sporadically; however, it remains the most frequently used method to monitor individuals for cardiac arrhythmias (De Asmundis et al., 2014). An event monitor, similar to a Holter, is routinely recommended to be worn for a longer period of time and used for rarer arrhythmias (Clinic, 2019). These are user friendly devices that start recording cardiac electrical activity when the patient has onset of arrhythmic symptoms (Clinic, 2019). Multiple ambulatory devices are now in practice that enable monitoring vital signs both in-hospital and in remote populations (Kulkarni et al., 2021a). *Trans*-telephonic transmitters on the other hand are a slight variation from other ambulatory devices and are not worn throughout the day but rather only during phone monitoring sessions (Clinic, 2019).

Non-invasive cardiac mapping is now extensively used in clinical practice (Dubois et al., 2015) to identify the sources of electrical disorders and guide catheter ablation of atrial arrhythmias (premature atrial beat, atrial tachycardia, AF), ventricular arrhythmias and ventricular pre-excitation (Wolff–Parkinson–White syndrome) (Dubois et al., 2015). In conjunction, echocardiography is often used when managing patients with congenital heart disease and supraventricular arrhythmias (Papadopoulos et al., 2018). It aids clinicians in making decisions about therapies for rhythm/rate control, cardiac ablations, and left atrial appendage closures (Papadopoulos et al., 2018).

Tilt table tests are another useful clinical modality for arrhythmia diagnosis in specific subgroups of patients presenting with syncope. Studies have demonstrated that patients with a positive head-up tilt table test are likely to exhibit arrhythmias (Hermosillo et al., 1999; Lj et al., 2010; Teodorovich and Swissa, 2016) and in particular sinus bradycardia, junctional escape rhythm or sinus arrest (Lj et al., 2010). A retrospective study of patients with a history of syncope, who had undergone head-up tilt table test with nitroglycerin or isoprenaline demonstrated a higher yield of a positive response, lower incidence of unwanted arrhythmias and better tolerability with nitroglycerin provocation compared to isoprenaline in patients with suspected neuro cardiogenic syncope (Prabhu et al., 2017). Yet, in recent years, the clinical applicability of this test has been questioned given the high false positive rates and the failure to establish an explicit cause of syncope making it incapable of guiding treatment (García-Civera et al., 2005; Kulkarni et al., 2020).

#### Novel Non-linear Dynamical Methods

Advances in data science techniques and the use of machine learning (ML) and deep learning (DL) have enabled the development of accurate models that can analyze large amounts of clinical data to classify cardiac rhythms and predict the onset of cardiac arrhythmias (Table 1). AI has shown its presence in cardiac electrophysiology since the 1970s in the form of automated ECGs (Krittanawong et al., 2020). Nonetheless, recent developments of expertly labeled data in large electronic databases have increased the value of AI in cardiac imaging and electrophysiology and has been put to use for enhanced prediction, response to therapy and characterization of health and diseases.

**TABLE 1:** Prediction of cardiac arrhythmias: summary of recent non-linear dynamical and artificial intelligence (AI) algorithms for arrhythmia classification and prediction.

Author/study	Year	Aim	Method	Efficacy
Jeong et al., 2021	2021	Prediction of VF	ANN, Feature extraction from HRV signals	Prediction accuracies of 88.18 and 88.64% at HRV data lengths of 10 and 20 s
Taye et al., 2019	2019	Prediction of VF	ANN, Feature extraction from HRV and QRS complex	72% (HRV) and 98.6% (QRS)
Shakibfar et al., 2019	2019	Prediction of short term risk of electrical storm	Random forest analysis of daily ICD summaries	Accuracy of 0.96 and AUC 0.80
Raghunath et al., 2021	2021	Identify patients at high-risk for new-onset AF	Deep neural network-based analysis of 12-lead ECGs	AUC 0.85, sensitivity of 69% and specificity of 81%
Vassilikos et al., 2011	2011	Prediction of PAF in patients with no history of structural heart disease.	Orthogonal ECG-based wavelet analyses of P waves	Larger energies of P-wave at X lead and larger left atrium were associated independently with >5 PAF episodes
Namarvar and Shahidi, 2004	2004	Prediction of arrhythmias	Wavelet analysis and SVM application to ECGs of 35 patients	Sensitivity of 92% and specificity of 75%
Bertsimas et al., 2021	2021	Arrhythmia classification	XGBoost machine learning algorithm	F1 scores (measure of test’s accuracy) in the range 0.93–0.99
Bundy et al., 2020	2020	Predict 5-year AF risk	Clinical variables in CHARGE-AF and variables extracted using random forest ML algorithm	C-statistic of 0.806
Heo et al., 2019	2019	Predict long-term outcomes in ischemic stroke patients	Deep neural network	AUC 0.88
Boon et al., 2016	2016	Predict PAF using short HRV segments and genetic algorithm	SVM to evaluate ECG signals	Prediction accuracy of 83.9%
Mohebbi and Ghassemian, 2012	2010	Prediction of PAF	HRV signal extraction and SVM-based classification	Sensitivity, specificity, and positive predictive value of 96.30, 93.10, and 92.86%, respectively
Costin et al., 2013	2013	Prediction of PAF	HRV analysis and morphologic variability of QRS complex	Prediction accuracy of 90% when using the methods in combination
Tiwari et al., 2020	2020	Identify patients at risk of 6-month incident AF	Analysis of electronic health records using Naïve Bayesian system	Optimal prediction of 6-month incident AF with AUC of 0.800 and F1 score of 0.110
Jin et al., 2009	2009	Detection of abnormal CVD conditions through a phone app	ECG recording and analysis to classify the patient in one of the arrhythmias	Prediction accuracy of 90%
Patel and Sengupta, 2020	2020	Machine learning applied on ECG	HRV segment analysis to predict SCD	Model predicted SCD with an accuracy of 84%, 4 min before the event
Golińska et al., 2020	2020	Prediction of the onset of arrhythmias in patients with ICDs	RR intervals from ICD patients and analyzed by random forest ML model	AUC 0.82
Lai et al., 2019	2019	Prediction of sudden cardiac death	Analysis of repolarization intervals and conduction-repolarization markers from 12 -lead ECGs using ML classifiers	Accuracy of 98.91% k-nearest neighbor, 98.70% (SVM), 98.99% decision tree, 97.46% Naïve Bayes, and 99.49% random forest in predicting sudden cardiac death 30 min before occurrence
Hill et al., 2020	2019	Assess cost effectiveness of targeted screening to identify patients with AF	Use of Markov AF disease progression model and hybrid screening decision tree	Patients needed to screen reduced from 534 per 1,000 to 61 per 1,000 patients using ML
Ong et al., 2012	2012	Risk stratification for cardiac arrest	Prospective observational study conducted on critically ill patients by analyzing the HRV segments in combination with age and vital signs and generating a ML score for risk stratification	AUC for ML scores in predicting cardiac arrest within 72 h was 0.781

*DL, deep learning; HF, heart failure; ANN, artificial neural network; SVM, support vector machine; ML, machine learning; AUC, area under curve; ICD, implantable cardioverter defibrillator; PAF, paroxysmal atrial fibrillation; HRV, heart rate variability; AF, atrial fibrillation; CVD, cardiovascular disease; CRT, cardiac resynchronization therapy.*

Many studies have shown ML methods to be superior over statistical-based models in predicting cardiovascular events (Patel and Sengupta, 2020; Bazoukis et al., 2021). Shakibfar et al. (2019) used a random forest ML model to analyze daily summaries of ICDs monitoring to predict electrical storms (arrhythmic syndrome). Using data from 19,935 patients, they demonstrated that short risk of arrhythmic syndrome could be successfully predicted using a ML model with an accuracy of 96%, significantly outperforming traditional logistic regression analysis. Another recent study found that after training a deep neural network with >1 million 12-lead resting EKGs, the model was able to predict new-onset AF within 1 year in patients with no history of AF with an area under the receiver operating characteristic curve of 0.85 (Raghunath et al., 2021). The DL model significantly outperformed both the extreme gradient boosting (XGBoost) (Chen and Guestrin, 2016) model using only age and sex as inputs and the CHARGE-AF (Cohorts for Aging and Research in Genomic Epidemiology) 5-year risk prediction model (Alonso et al., 2013). These promising studies demonstrate the utility of AI models to predict cardiac arrhythmias, while also highlighting the importance of labeling clinical data and outcomes, and harmonizing data across institutions for improved diagnostics.

### Prevention and Control of Atrial Arrhythmias

#### Traditional Clinical Methods

Clinical management of arrhythmia begins by formulating a definitive diagnosis. A goal directed treatment approach for the specific type of arrhythmia is mandatory. Management strategies have been structured based on the use of antiarrhythmic drugs or ICDs or both. Even with recent advances, pharmacological therapy has remained as mainstay unless contraindicated. Traditionally, many studies conducted to study the risks and benefits of the currently available antiarrhythmic drugs have shown that except for Amiodarone and Sotalol, other classes of antiarrhythmic medications are not sufficiently effective (Auer et al., 2002). Amiodarone and Sotalol are primarily classified as class III antiarrhythmic drugs which act by blocking the outward potassium channels responsible for the repolarization of the heart. Both are known to reduce the risk of ICD shock from ventricular as well as atrial arrhythmia (Auer et al., 2002; John et al., 2012). Though Amiodarone is known to be better tolerated in comparison to the other drugs, its use and overall efficacy is limited due to its known side effects affecting the cardiac, ophthalmologic, thyroid, and hepatic systems (Auer et al., 2002; Florek and Girzadas, 2018; Lantz and Agarwal, 2021). Thus, ongoing pharmacological interventional studies are now focused on the selective ion channels [e.g., atrial I(Na), I(Kur) and I(K, ACh)], pathology-selective ion channels [constitutively active I(K, ACh), TRP channels], ischemia-uncoupled gap junctions, proteins related to malfunctioning intracellular Ca(2+) homeostasis [e.g., “leaky” ryanodine receptors, overactive Na(+), Ca(2+) exchanger] or risk factors for arrhythmias (“upstream” therapies) (Ravens, 2010).

In addition, catheter ablation is considered the mainstay therapy for drug-refractory AF and flutter (Hindricks et al., 2021). It has been an important part of the treatment protocol for a wide spectrum of atrial arrhythmias (Lee et al., 2012). Focal atrial tachycardia and typical atrial flutter is treated with catheter ablation as a first line with an efficacy of over 90%. Furthermore, ablation is sought to provide highly effective palliation and improved quality of life in patients who have had previous atrial surgery or complex congenital heart conditions and are hence, prone to developing late flutter recurrence or AF (Lee et al., 2012). In patients with persistent AF, the role of catheter ablation continues to evolve, as does our understanding of the crucial underlying mechanisms (Lee et al., 2012).

Atrial arrhythmias are also often controlled traditionally by overdrive pacing. The Pacemaker Atrial Fibrillation Suppression study performed a randomized, multicentre investigation of the effects of the third generation anti-atrial fibrillation pacemaker algorithms in patients with paroxysmal AF and demonstrated that the rate-soothing algorithm by atrial overdrive pacing decreased premature atrial contractions-initiated AF (Sulke et al., 2007). Similarly the ADOPT (Atrial Dynamic Overdrive Pacing Trial) study, conducted in patients with symptomatic paroxysmal or persistent AF and sinus node dysfunction demonstrated that overdrive atrial pacing with the AF Suppression Algorithm (St. Jude Medical Cardiac Rhythm Management Division, Sylmar, CA, United States) decreased symptomatic AF burden significantly in patients with sick sinus syndrome and AF (Carlson et al., 2003).

Implantable pacemakers have been a breakthrough in preventing atrial arrhythmias by using specific atrial pacing algorithms (Dimmer et al., 2003). Various studies have been done in the past which have shown that majority of the paroxysmal AF episodes are initiated by bradycardias, premature atrial complexes (PAC) or immediate reinitiation of AF, and multiple pacing modalities have been developed to prevent AF recurrences (Hoffmann et al., 2000; Attuel et al., 2003; Yang et al., 2003). These algorithms primarily rely on the classification of atrial events by the pacemakers and work either by responding to triggers or by continuous overdrive atrial pacing.

Commonly utilized arrhythmia control algorithms currently in clinical practice include pace conditioning, PAC suppression, post-PAC response, post-exercise response, post-AF response and rate soothing (Mitchell and Sulke, 2004). The post AF response algorithm is shown to prevent tachyarrhythmias which occur soon after termination of the previous episode due to immediate reinitiation of AF. After confirming the termination of an atrial tachycardia episode, the algorithm increases the pacing rate to the programmed response rate of 70–100 bpms for the next 600 beats and decays slowly until sinus rhythm is detected or a lower rate limit is achieved. Pace conditioning works similar to the dynamic atrial overdrive algorithm in St. Jude Medical (Sylmar, CA, United States) pacemakers, atrial preference pacing in Guidant (St. Paul, MN, United States) pacemakers, the DDD+ algorithm in Biotronik (Berlin, Germany) pacemakers and atrial preference pacing in Medtronic (Minneapolis, MN, United States) pacemakers. It works by adjusting the atrial pacing rate to just above the underlying intrinsic rhythm in a way that the atrium is paced for almost 95% of time. PAC suppression works by increasing the heart rate on detection of PACs in order to reduce incidence of PACs. The pacing rate is increased by 15 bpm above the physiological rate of 600 beats on detection of PACs. This increased rate is kept stable for a period of time and any PAC detected in this stable phase does not lead to further increase in the rate. After the stable period, the atrial rate decays by 1 bpm every 16 beats.

Increased vagal tone and consequent bradycardia in athletes can precipitate AF. The post-exercise pacing algorithm works by preventing rapid drop in heart-rate after exercise by enabling a post-exercise rate. During exercise, the post-exercise pacing rate slowly rises to 90% of the physiological rate which is dependent on the difference between the two rates. When the heart rate suddenly decreases after exercise, the pacemaker paces at the post-exercise pacing rate. The Medtronic “atrial rate stabilization” works using the principle of post PAC response. Post PAC response prevents pauses after PACs by controlling the atrial rate in the two beats after a PAC. Finally, the rate soothing technique works similar to pace conditioning except that the heart rate is not substantially increased but rather atrial tachycardias are prevented by overdrive pacing the atrium at a rate that is only just above the sinus rate.

Despite these promising advances in arrhythmia control algorithms, the clinical efficacy of the current conventional methods including overdrive pacing remain questionable due to false positives and adverse effects on device battery life (Camm et al., 2007; de Voogt et al., 2007; Hohnloser et al., 2012), warranting the need for novel approaches to suppress abnormal cardiac rhythms. Furthermore, though pharmacological treatment along with interventional techniques is the mainstay of cardiac arrhythmia management, studies conducted consistently demonstrated a strong association between lifestyle interventions and arrhythmia prevention. Obesity (defined as BMI ≥ 30 kg/m^2^) is documented to be a strong risk factor and associated with the incidence of AF (Chung et al., 2020). It has been demonstrated that a patient who lost and maintained the loss of ≥10% of their body weight enjoyed a sixfold arrhythmia-free likelihood compared with those who lost <3% or gained weight (Chung et al., 2020). Regular aerobic exercise as well as moderate intensity of physical activity is highly recommended (Chung et al., 2020). A randomized control trial of 28 patients with persistent AF compared a non-exercise control with 2 months of moderate-intensity continuous training (Chung et al., 2020). The study demonstrated the latter subjects showed reduced AF-related symptoms along with improved quality of life and exercise capacity.

#### Autonomic Modulation of Cardiac Arrhythmias

The cardiovascular system is innervated by the central nervous system, the extrinsic intrathoracic ganglia and the intrinsic cardiac autonomic nervous system (CANS). Autonomic and neurohormonal reflexes regulate the CANS. CANS balance is maintained via baroreflex which maintains the blood pressure and after load on the heart achieved through sympathetic-parasympathetic balance (Benarroch, 2008) while the neurohormonal feedback is achieved by the renin-angiotensin aldosterone system (Goldsmith, 2004). For over 20 years autonomic modulation has been used for neurological diseases like epilepsy (Ben-Menachem, 2002) and psychiatric diseases like depression (Bajbouj et al., 2010). In recent years, studies have shown that autonomic modulation can play a role in treatment of cardiovascular diseases like heart failure and arrhythmias (Florea and Cohn, 2014; Goldberger et al., 2019; Stavrakis et al., 2020a; Kulkarni et al., 2021b).

Autonomic modulation can be used for the treatment of atrial as well as ventricular arrhythmias and can be achieved by vagal nerve stimulation, tragus stimulation, renal denervation, spinal cord stimulation, baroreceptor stimulation or left cardiac sympathetic denervation (Stavrakis et al., 2020a). Even though treatment of cardiac arrhythmias through automatic modulation has proven results preclinically, large randomized clinical trials have shown mixed results (Premchand et al., 2014; Zannad et al., 2015; Gold et al., 2016; Stavrakis et al., 2020b), highlighting the need for optimized biomarkers enabling appropriate patient selection. Yet, recent studies have demonstrated that vagal stimulation can modulate cardiac electrophysiological properties (Kulkarni et al., 2018b; Lee et al., 2018) including both atrial and ventricular alternans (Kulkarni et al., 2021b,c) as well as reduce AF burden (Qin and Singh, 2020; Stavrakis et al., 2020b).

## Management of Ventricular Arrhythmias

### Prediction Modalities

#### Traditional Clinical Methods

Various ECG ventricular repolarization markers (Tse and Yan, 2017) such as QT interval, the time interval from the start of the QRS complex to the end of the T wave (Castro-Torres et al., 2015), QTc, corrected QT interval normalized to the underlying heart rate, QTd, the dispersion of the QT interval, Tp-e, the time duration from the peak to the end of the T wave (Gürdal et al., 2017), Tp-ed, the dispersion of the Tp-e interval (Castro-Torres et al., 2015) and Tp-e/QT, the ratio between the Tp-e and QT intervals, have been used to predict the prevalence of cardiac arrhythmias. QTd is commonly used to denote the variability of ventricular repolarization. It has been proposed that the higher the QTd, the higher the risk for an individual to be predisposed to ventricular arrhythmias (Castro-Torres et al., 2015). Similarly, the Tp-e interval marks the transmural cardiac repolarization and is an important risk indicator for ventricular arrhythmias (Castro-Torres et al., 2015; Tse et al., 2017). It has been shown to be a more accurate marker to predict cardiac arrhythmias than QTc or QTd (Castro-Torres et al., 2015). Using the Tp-e marker, individuals with Brugada syndrome were found to be at a greater risk for ventricular arrhythmias than the healthy control group (Castro-Torres et al., 2015; Maury et al., 2015), while a meta-analysis of 155,856 patients demonstrated that prolonged Tp-e interval was a significant predictor of arrhythmic or mortality outcomes (Tse et al., 2017). The ratio between Tp-e and QT is a novel method to detect cardiac arrhythmias and is suggested to have greater accuracy than other markers as it has very few to no variations between 60 and 100 beats/min and hence, relatively independent of heart rate (Castro-Torres et al., 2015). Studies have shown that reduced coronary flow is positively correlated to the prolonged Tp-e interval and Tp-e/QT ratio (Zehir et al., 2015). Furthermore, prolonged Tp–e interval and increased Tp-e/QTc ratio have been shown to be independently associated with ventricular arrhythmic events in hypertrophic cardiomyopathy patients (Akboğa et al., 2017). Yet, while ventricular repolarization markers have been found to be useful in predicting malignant cardiac arrhythmias in many medical conditions, comprehensive investigative studies focused on their interpretation, reproducibility, robustness against interpatient variability and clinical utility are still warranted (Castro-Torres et al., 2015; Tse and Yan, 2017).

Another common clinical arrhythmia diagnostic method are stress tests, typically performed on individuals whose arrhythmias are adrenergically-driven and thus present or worsen with exercise (Clinic, 2018). Exercise is a known arrhythmogenic that may trigger ventricular premature beats, VT, VF or extrasystoles in patients with coronary heart disease (Jelinek and Lown, 1974). Similarly, they are an extremely important modality to detect occult arrhythmias (Jelinek and Lown, 1974). However, there are many limitations that make stress tests an obsolete testing modality (Jelinek and Lown, 1974). Many studies have shown that stress tests may present false positive results in asymptomatic patients (Coplan and Fuster, 1990). Furthermore, individuals with coronary artery disease are less likely to complete the stress test due to stress angina, thus making it challenging to detect the arrhythmia (Jelinek and Lown, 1974) and limiting the use of stress tests in clinical diagnosis (Coplan and Fuster, 1990). EP study, is a minimally invasive procedure that uses catheters to record the electrical activity of the heart (Stouffer et al., 2001) and is sometimes used to stratify arrhythmic risk and propensity for SCD, especially in patients with a left ventricular ejection fraction greater or equal to 35% (Hilfiker et al., 2015). More recently, non-invasive cardiac mapping has been shown to be useful in identifying sources of premature ventricular complexes and VT (Dubois et al., 2015) and successfully guiding catheter ablation, reducing the need for invasive mapping (Cakulev et al., 2013; Jamil-Copley et al., 2014).

#### Novel Non-linear Dynamical Methods

Non-linear dynamical approaches have long been utilized for predicting the onset of cardiac arrhythmias and the transition from steady state to abnormal electrical activity. Wenckebach phenomenon and parasystole were the first couple of arrhythmias where non-linear dynamics were used for systematic investigation (Krogh-Madsen and Christini, 2012). Recently, using preclinical studies, it has been shown that when the heart is perturbed from its steady state by electrical stimulation, only a small number of dominant eigenvalues are associated with it and principal component analysis enables evaluating the dominant eigenvalues for prediction of alternans (Jackson, 2005; Petrie and Zhao, 2012), a beat-to-beat alternation in the action potential duration of cardiac myocytes which manifests as oscillations in the T-wave morphology or duration on the ECG. During alternans, the heart transitions from a 1:1 stable rhythm to a 2:2 alternating pattern by means of a period doubling bifurcation, which occurs when a negative eigenvalue approaches – 1 (Strogatz, 1994; Li and Otani, 2003; Otani et al., 2005). Using *ex vivo* whole heart experiments, it was demonstrated that the onset of localized spatial alternans can be successfully predicted by applying principal component analysis to 2-dimentional cardiac optical mapping data (Kakade et al., 2013). Furthermore, the application of classical non-linear dynamical theory to investigate the bifurcation to abnormal cardiac rhythms, has enabled the classification of the transition from steady state to alternans. Preclinical optical mapping experiments on isolated whole rabbit hearts has demonstrated that the onset of alternans predominantly occurs through a smooth bifurcation (Kulkarni et al., 2015), which provides insights into a systemic understanding of cardiac dynamics and offers potential modalities for the prevention and control of the cardiac system based on non-linear approaches.

Another important biomarker that has shown to aid detection and prediction of VT/VF is heart rate variability (HRV), an inherent variation in the time interval between consecutive heart beats. Clinically, low HRV is a prognostic marker of an underlying cardiac functional abnormality (Jeong et al., 2021). Application of non-linear systems theory based methods to HRV markers have provided additional prognostic information compared to traditional HRV measures (Huikuri et al., 2003). In a novel study combining the benefits of AI with clinical HRV measures for cardiac arrhythmia diagnosis, Jeong et al. (2021) predicted the occurrence of VF with high accuracies of 88.18% and 88.68% using an artificial neural network which was trained using features from HRV signals with optimal data lengths of 10 s and 20 s and the forecast time period being 0 s.

Another study by Taye et al. (2019) compared the accuracy of applying DL techniques and predicting cardiac arrhythmias using HRV vs. QRS complex features. They trained two artificial neural networks and tested one each with features derived from either HRV measures or the QRS complex and found the former had 72% prediction accuracy while the latter had 98.6%, hence highlighting the QRS complex’s important role in accuracy improvement of VF prediction. They were able to predict onset of VF 30 s before occurrence using features extracted from 120 s long ECG and HRV signals. Prediction models dependent on QRS complexes are based on the principle of reentrant circuits. Electrical activity in the heart starts to change when the ventricles begin making reentrant waves due to ectopic focus hence changing the QRS complex and prompting the hypothesis that QRS shape features alone could be efficient VF predictors (Taye et al., 2019). In alignment with this theory, Bertsimas et al. (2021) designed a prediction system based on the QRS complex with the potential to be integrated in wearables that could predict cardiac arrhythmias and distinguish between AF, tachycardia, bradycardia or noise within 30 ms, from an ECG signal shorter than a minute.

### Prevention and Control of Ventricular Arrhythmias

#### Traditional Clinical Methods

Overactivity of the sympathetic nervous system has been associated to ventricular tachyarrhythmias and sudden death (Singh, 2005). Beta-blockers can effectively suppress ventricular ectopic beats and arrhythmias, can be used to control rapid ventricular activation due to rapid and irregular atrial firing during AF as well as to prevent SCD in a wide range of cardiac diseases (Haverkamp et al., 1990; Poole-Wilson et al., 2003; Grandi and Ripplinger, 2019). Beta-blockers, as a class of compounds, have the essential pharmacologic function of counteracting the actions of competitive adrenergic receptors (Singh, 2005; Grandi and Ripplinger, 2019). The net clinical effects of various beta-receptor blockers, however, may differ quantitatively due to differences in related intrinsic sympathomimetic agonism and intrinsic potency for binding to beta-receptors (Grandi and Ripplinger, 2019). Evidence supports the idea that the antiarrhythmic effects of certain beta-receptor blockers, such as Carvedilol and Metoprolol, extend beyond the ventricular tissue to encompass atrial cells and help maintain sinus rhythm in patients with AF, particularly when combined with potent antifibrillatory agents like Amiodarone (Haverkamp et al., 1990). Side effects are typically dose-related; optimal and lowest effective beta-blocker doses are critical in limiting unwanted effects (Haverkamp et al., 1990). A larger use of beta-blockers as antiarrhythmic medicines in the future, possibly in combination with class I or III therapy, is feasible.

However, with the evolving advancements, the management of cardiac arrhythmias has been changing significantly over the years. ICDs are designed to monitor the heart constantly, identify malignant ventricular tachyarrhythmias and deliver anti-tachycardia pacing and/or an electrical countershock to restore normal rhythm (Mirowski, 1985). A meta-analysis study conducted showed that defibrillators when used as a primary or secondary prevention strategy is highly effective (Lee et al., 2003). The study demonstrated a relative risk reduction of 50% and 45% for arrhythmic death among the secondary and primary prevention trials, respectively. In addition ICDs have been shown to successfully terminate lethal arrhythmias and enable substantial improvement in survival in high-risk patients (Mirowski, 1985). Furthermore, ICDs have enabled a 20% relative risk reduction in all-cause mortality and a 33% reduction in arrhythmic mortality in comparison to pharmacological treatment with Amiodarone (Connolly et al., 2000).

Low energy shocks have been proposed to be painless and shown efficacy in terminating ventricular tachyarrhythmias in large animal models (Jackman and Zipes, 1982; Moreno et al., 2021). Furthermore, it has been postulated that autodecremental overdrive pacing and low-energy cardioversion have similar efficacy and acceleration rates (Bardy et al., 1993). In this study comparing anti-tachycardia pacing with low-energy cardioversion in 24 patients, VT was repeatedly terminated in 18 out of 24 patients by low-energy cardioversion, while 5 out of 24 patients saw VT accelerated to faster VT or VF, overall demonstrating similar response as with over drive pacing (Bardy et al., 1993). However, more recent human studies have shown low-energy shocks to be less effective than and more arrhythmogenic than high energy shocks (Strik et al., 2020), warranting further investigation of the clinical utility of this approach.

For non-ischemic VTs, it has been evidenced that catheter ablation is a reasonable first-line therapy as there has been a recorded 80–90% success rate in an experienced clinical center (Hall and Todd, 2006). With the advent of the modern non-fluoroscopic mapping system, catheter ablation is now possible in patients with or without structural heart disease. With the use of catheter ablation becoming widely available, a study demonstrated a curable rate of 90% for atrial flutter patients with severe symptoms (Hall and Todd, 2006). Radiofrequency ablation has evolved as a treatment of choice for idiopathic VT arising from the right ventricular outflow tract, demonstrating success rates between 75% and 100%, and for idiopathic left ventricular (fascicular) VT success varies from 50% to 90% (Nakagawa et al., 1993; Wellens and Smeets, 1993; O’donnell et al., 2003).

Another treatment modality for VT suppression is ventricular overdrive pacing, wherein stimuli are applied from the right ventricular apex during tachycardia at a rate that is little faster than the tachycardia cycle length until the paced QRS stabilizes (Kneller, 2015). The long-term overdrive suppression of refractory ventricular arrhythmias is reported to be frequently successful, more so if the heart rates are slow, but also in patients with normal heart rates (Johnson et al., 1974).

#### Novel Pacing Modalities

Besides the traditional methods of arrhythmia prevention, more recently, novel pacing techniques have reported promising anti-arrhythmic results in *in silico* and preclinical experimental studies (Table 2). Given the intrinsic variability in cardiac beat-to-beat intervals (HRV), many studies have investigated the effect of incorporating stochasticity in pacing on cardiac arrhythmogenesis (Dvir and Zlochiver, 2013; Kulkarni et al., 2016; Prudat et al., 2016; Wilson and Ermentrout, 2017). Cardiac alternans, which is suggested to be a precursor to fatal cardiac arrhythmias such as VT and VF, is a complex dynamical phenomenon originating at the cellular level as a result of instabilities in ion current dynamics (voltage-driven alternans) or intracellular calcium [Ca(2+)] cycling (Ca(2+)-driven alternans) (Prudat et al., 2016; Kulkarni et al., 2019). In a recent study, Prudat et al. (2016) demonstrated the utility of stochastic pacing in discriminating between voltage-gated and Ca(2+)-driven alternans, providing insights into the onset of cardiac rhythm instabilities. The influence of stochastic pacing on cardiac arrhythmogenicity was further illustrated by Dvir and Zlochiver (2013) using one-dimensional and two-dimensional ventricular models. They demonstrated that increased stochasticity in pacing promoted anti-arrhythmic effects and eliminated discordant action potential duration alternans (Dvir and Zlochiver, 2013). Their results suggest that the stabilization of cardiac electrical conduction can be improved by adding specific stochasticity to the programmed pacing sequence which inhibits the initiation of the arrhythmic events instigated by conduction blocks or wave breaks (Dvir and Zlochiver, 2013).

**TABLE 2:** Prevention and control of cardiac arrhythmias: summary of novel pacing techniques for arrhythmia suppression.

Author/study	Year	Aim	Method	Inference
Prudat et al., 2016	2016	Examine utility of stochastic pacing to discriminate between voltage-driven and Ca(2+)-driven alternans	Stochastic pacing	There is a possibility to discriminate between voltage-driven and Ca(2+)-driven alternans with a sensitivity and specificity >80%
Merchant et al., 2020	2020	To develop a closed-loop system capable of detecting T-wave alternans in real-time and delivering pacing stimuli	Real-time closed-loop suppression of repolarization alternans using pacing during the ARP	Suppressing alternans using R-wave triggered pacing during the ARP reduces arrhythmia susceptibility
Dvir and Zlochiver, 2013	2013	Characterize the effects of stochastic pacing on ventricular tissue arrhythmogenic predictors of restitution slopes and APD alternans	Geometrical and biophysical model, stochastic pacing	Stochastic pacing reduces the risk of cardiac arrhythmias
Wilson and Ermentrout, 2017	2017	Investigate the anti-arrhythmic properties of stochastic pacing	Stochastic pacing	Stochastic pacing rates can be antiarrhythmic and inhibit the formation of discordant alternans
Jordan and Christini, 2004	2004	Control action potential duration alternans using electrical stimulation	Adaptive diastolic interval control algorithm	Adaptive diastolic interval pacing controls alternans in single cell model but exhibits limited spatial control of alternans in 1D model
Kulkarni et al., 2018a	2018	Validate the anti-arrhythmic effects of constant diastolic interval pacing	A new closed loop system which detects T-waves from real-time ECG and applies stimuli after predefined constant diastolic intervals on a beat-by-beat basis	Maintaining a constant diastolic interval on an every beat basis prevents the spatiotemporal onset of voltage driven restitution dependent alternans in isolated whole rabbit hearts
Sridhar et al., 2013	2013	Suppression of alternans using *ex vivo* and *in silico* models	Alternating-period-feedback stimulations	This method is more robust to noise than previous alternans reduction techniques based on fixed point stabilization
Zagrodzky et al., 2001	2001	To assess the effect of biventricular pacing on the inducibility of sustained monomorphic VT in patients with coronary artery disease	Biventricular pacing	Acute biventricular pacing decreases the inducibility of sustained monomorphic VT in patients with ischemic cardiomyopathy
Kowal et al., 2004	2001	To evaluate in a prospective randomized fashion the electrophysiologic effects of acute biventricular pacing	Biventricular pacing	Biventricular pacing significantly reduces susceptibility to VT compared to the RV pacing

*ARP, absolute refractory period; VT, ventricular tachycardia; RV, right ventricular.*

Similar anti-arrhythmic effects of stochastic pacing were demonstrated by Wilson and Ermentrout (2017) wherein the formation of discordant alternans was inhibited in both one and two dimensional models. However, contradictory effects of stochastic pacing were reported in other studies wherein the onset of alternans was still present despite incorporating variability in pacing intervals (McIntyre et al., 2014; Kulkarni et al., 2016, 2018a). These recent studies suggested that pacing, with or without stochasticity can be pro-arrhythmic under certain conditions especially when the mean duration between consecutive stimuli is maintained constant, namely during periodic pacing.

A novel pacing technique that eliminated the constraints of periodic pacing by controlling the diastolic interval, was investigated by Jordan and Christini (2004). Their adaptive diastolic interval pacing technique demonstrated control of alternans in a one dimensional Purkinje fiber *in silico* study, but exhibited limited spatial control (Jordan and Christini, 2004). However, more recently the anti-arrhythmic effects of maintaining a constant diastolic interval on an every beat basis was demonstrated using *ex vivo* optical mapping experiments on isolated whole rabbit hearts (Kulkarni et al., 2018a). Kulkarni et al. (2018a) developed a novel closed loop system capable of detecting T-waves from ECG in real-time, enabling the successful implementation of constant diastolic interval pacing, which was shown to be anti-arrhythmic and prevented the spatio-temporal onset of alternans in the heart. While the utility of controlling the diastolic interval on a beat-by-beat basis in preventing voltage driven restitution dependent alternans has been effectively demonstrated, its efficacy in suppressing arrhythmias originating from metabolic and Ca(2+) cycling instabilities remains to be investigated (Kulkarni et al., 2018a,2021d).

Another anti-arrhythmic pacing technique, namely the alternating-period-feedback protocol, has been proposed which demonstrably reduced the presence of alternans in isolated whole heart experiments (Sridhar et al., 2013). The protocol aimed at suppressing alternans by applying small perturbations to the pacing cycle length and was proposed to be more robust than previous alternans reduction techniques offering promising implications for experimental investigations into its clinical utility for arrhythmia prevention.

To treat patients with ventricular dysfunction, biventricular pacing (BV) has been an effective modality even though electrophysiologic effects of BV pacing remain poorly understood (Zagrodzky et al., 2001). A study by Zagrodzky et al. (2001) assessed the effect of BV pacing on the inducibility of sustained monomorphic VT in patients with coronary artery disease and demonstrated a 60% reduction in inducibility with BV pacing as compared to single-site right ventricular pacing. Similar findings were reported by other studies over the years supporting the hypothesis that BV pacing reduces the frequency of ventricular arrhythmias, however, the mechanism of arrhythmia suppression remains unclear (Kowal et al., 2004).

#### Dynamic Control of Arrhythmias

As the development of arrhythmias is a dynamic instability shift from sinus rhythm, many dynamical approaches are being investigated to control these instabilities and prevent the occurrence of arrhythmias. Dynamic R-wave triggered pacing during the absolute refractory period has been shown to suppress spontaneous repolarization alternans, a pattern of ventricular repolarization that repeats on every other beat basis, which has been seen to be closely associated with VT/VF (Armoundas et al., 2013; Merchant et al., 2020). This novel pacing modality has been shown to suppress cardiac alternans *in vivo* and under diseased conditions by delivering subthreshold stimuli during the absolute refractory period to modulate ventricular repolarization, thus offering a potential technique to improve the current ICDs by preventing the onset of ventricular arrhythmias. Cardiac alternans has also been shown to be controlled by mechanical perturbation, which can alter the electrical activity of tissue by mechano-electric coupling, in *in silico* studies (Dubljevic and Christofides, 2008; Hazim et al., 2015).

Chaos control based non-linear dynamical approaches have shown promising results in controlling arrhythmias in *in vitro* and *in silico* studies (Christini et al., 1999, 2001; Kulkarni and Tolkacheva, 2021). Christini et al. (1999, 2001) demonstrated the successful termination of pacing-induced period-2 atrioventricular-nodal conduction alternans using non-linear dynamical control by stabilizing the underlying unstable steady-state conduction in 52/54 control attempts in five patients. Chaos control techniques have been shown to modulate human cardiac electrophysiological dynamics and successfully prevent cardiac arrhythmias by stabilizing unstable rhythms through small time continuous perturbations aimed at restoring the heart to a steady state, which has potential to be incorporated into pacemakers (Garfinkel et al., 1992, 1995; Christini et al., 2001; Ferreira et al., 2011).

## Future Perspectives: Bridging the Gap Between Basic and Clinical Science

As we embark into 60 years of cardiac pacing, the field has seen significant technological advancements and continued progress. Cardiac stimulation strategies have registered significant benefits with reductions in HF hospitalization, AF and mortality. However, as summarized in this review, there are several shortcomings in the existing treatment strategies and many current pacing devices face challenges of false positives, suboptimal accuracy of predicting the onset of abnormal rhythms and low efficacy in suppressing ongoing arrhythmic events. Novel pacing strategies that are under research and development although show promise, there exist several challenges to bring these technologies into clinical practice that warrants further research for testing and validating the safety and efficacy of these newer modalities.

Innovative approaches to predict the onset of cardiac arrhythmias as well as control abnormal rhythms offer huge promise in reducing the arrhythmic burden in preclinical and *in silico* experiments. Yet focused studies investigating their robustness and accuracy are required that can show the pathway toward clinical translation. Firstly, non-linear dynamical approaches and AI based strategies offer computational challenges in pacing devices which directly demands longer battery life. Limited battery life, warranting device replacements, lead extractions and related complications have challenged the pacing field significantly. Researchers should be wary of this important limitation and consider concurrent investigations of both cardiac pacing and battery technology together. Battery-less, solar-powered cardiac pacing offers significant promise, however, it has fundamental challenges to be addressed in terms of climate, energy harvesting and early warning of low battery levels.

Secondly, the advent of AI in cardiac dynamics is showing a steady increase in both prediction and control strategies of cardiac pacing. However, to date, there are no ML models that have been highly robust and accurate when dealing with interpatient and inter-institutional variability, hence questioning their translatability into a usable universal diagnostic tool. Several researchers have used millions of ECG datasets to develop prediction models, which are mostly black box applications with no novel insights into the physiological mechanisms of arrhythmia etiology that significantly hamper clinical translation. The gap between basic and clinical science is inadvertently increasing due to this approach and more interpretable ML models with physiological significance are required to bridge this gap. Therefore, a strong synergistic effort between basic scientists for developing and validating novel pacing strategies, cardiologists as well as pacemaker design industries is imperative to advance this important clinical need that can play a crucial role in cardiac patient healthcare.

## Conclusion

Novel pacing modalities for control and suppression of cardiac arrhythmias have shown promising results in translational preclinical studies. Applying non-linear dynamical approaches combined with advances in AI has opened the doors for investigating volumes of patient data and extracting relevant information for aiding clinical diagnostics and decision making. Predicting the onset of arrhythmias and the transition from steady to unstable cardiac rhythms has been a long standing challenge which newer non-linear methods have shown promising efficacy in addressing. However, the clinical adaptation of AI and dynamical pacing strategies would require a comprehensive framework that accounts for a reliable, accountable and secure diagnostic system, encouraging the active participation by researchers, engineers, technicians and physicians alike. While the novel pacing modalities and predictive algorithms discussed here are a promising first step for advancing cardiac arrhythmia management strategies, a thorough clinical investigation and physician assessment are necessary for the successful translation of these techniques into a diagnostic approach. Such an integrative approach combining medicine and engineering principles holds promise in pushing traditional boundaries and translating preclinical findings into clinically viable devices for cardiac arrhythmia prediction, prevention and control.

## Author Contributions

All authors listed have made a substantial, direct, and intellectual contribution to the work, and approved it for publication.

## Conflict of Interest

The authors declare that the research was conducted in the absence of any commercial or financial relationships that could be construed as a potential conflict of interest.

## Publisher’s Note

All claims expressed in this article are solely those of the authors and do not necessarily represent those of their affiliated organizations, or those of the publisher, the editors and the reviewers. Any product that may be evaluated in this article, or claim that may be made by its manufacturer, is not guaranteed or endorsed by the publisher.

## References

[B1] Akboğa M. K. Balcı K. G. Yılmaz S. Aydın S. Yayla Ç Ertem A. G. (2017). Tp-e interval and tp-e/qtc ratio as novel surrogate markers for prediction of ventricular arrhythmic events in hypertrophic cardiomyopathy. *Anatolian J. Cardiol.* 18:48. 10.14744/AnatolJCardiol.2017.7581 28315570 PMC5512198

[B2] Alonso A. Krijthe B. P. Aspelund T. Stepas K. A. Pencina M. J. Moser C. B. (2013). Simple risk model predicts incidence of atrial fibrillation in a racially and geographically diverse population: the charge-af consortium. *J. Am. Heart Assoc.* 2:e000102. 10.1161/JAHA.112.000102 23537808 PMC3647274

[B3] Armoundas A. A. Weiss E. H. Sayadi O. Laferriere S. Sajja N. Mela T. (2013). A novel pacing method to suppress repolarization alternans *in vivo*: implications for arrhythmia prevention. *Heart Rhythm.* 10 564–572. 23274372 10.1016/j.hrthm.2012.12.026

[B4] Arora V. Suri P. (2021). Physiological pacing: a new road to future. *Indian J. Clin. Cardiol.* 2 32–43. 10.1093/milmed/usy171 30020511 PMC6751385

[B5] Attuel P. Danilovic D. Konz K. H. Brachmann J. Allaf D. E. Löscher S. (2003). Relationship between selected overdrive parameters and the therapeutic outcome and tolerance of atrial overdrive pacing. *Pacing Clin. Electrophysiol.* 26 257–263. 10.1046/j.1460-9592.2003.00028.x 12687824

[B6] Auer J. Berent R. Eber B. (2002). Amiodarone in the prevention and treatment of arrhythmia. *Curr. Opin. Investig. Drugs* 3 1037–1044. 12186264

[B7] Bajbouj M. Merkl A. Schlaepfer T. E. Frick C. Zobel A. Maier W. (2010). Two-year outcome of vagus nerve stimulation in treatment-resistant depression. *J. Clin. Psychopharmacol.* 30 273–281. 20473062 10.1097/JCP.0b013e3181db8831

[B8] Bardy G. H. Poole J. E. Kudenchuk P. J. Dolack G. L. Kelso D. Mitchell R. (1993). A prospective randomized repeat-crossover comparison of antitachycardia pacing with low-energy cardioversion. *Circulation* 87 1889–1896. 10.1161/01.cir.87.6.1889 8504501

[B9] Bazoukis G. Stavrakis S. Zhou J. Bollepalli S. C. Tse G. Zhang Q. (2021). Machine learning versus conventional clinical methods in guiding management of heart failure patients—a systematic review. *Heart Fail. Rev.* 26 23–34. 32720083 10.1007/s10741-020-10007-3 PMC7384870

[B10] Benarroch E. E. (2008). The arterial baroreflex: functional organization and involvement in neurologic disease. *Neurology* 71 1733–1738. 10.1212/01.wnl.0000335246.93495.92 19015490

[B11] Ben-Menachem E. (2002). Vagus-nerve stimulation for the treatment of epilepsy. *Neurology* 1 477–482. 12849332 10.1016/s1474-4422(02)00220-x

[B12] Bertsimas D. Mingardi L. Stellato B. (2021). Machine learning for real-time heart disease prediction. *IEEE J. Biomed. Health Inform.* 25 3627–3637. 10.1109/jbhi.2021.3066347 33729960

[B13] Bhavnani S. P. Kluger J. Coleman C. I. White C. M. Guertin D. Shafi N. A. (2010). The prognostic impact of shocks for clinical and induced arrhythmias on morbidity and mortality among patients with implantable cardioverter-defibrillators. *Heart Rhythm.* 7 755–760. 10.1016/j.hrthm.2010.02.039 20211275

[B14] Boon K. H. Khalil-Hani M. Malarvili M. Sia C. W. (2016). Paroxysmal atrial fibrillation prediction method with shorter hrv sequences. *Comput. Methods Prog. Biomed.* 134 187–196. 10.1016/j.cmpb.2016.07.016 27480743

[B15] Bundy J. D. Heckbert S. R. Chen L. Y. Lloyd-Jones D. M. Greenland P. (2020). Evaluation of risk prediction models of atrial fibrillation (from the multi-ethnic study of atherosclerosis [mesa]). *Am. J. Cardiol.* 125 55–62. 10.1016/j.amjcard.2019.09.032 31706453 PMC6911821

[B16] Cakulev I. Sahadevan J. Arruda M. Goldstein R. N. Hong M. Intini A. (2013). Confirmation of novel noninvasive high-density electrocardiographic mapping with electrophysiology study: implications for therapy. *Circulation* 6 68–75. 10.1161/CIRCEP.112.975813 23275263

[B17] Camm A. Sulke N. Edvardsson N. Ritter P. Albers B. Ruiter J. (2007). Conventional and dedicated atrial overdrive pacing for the prevention of paroxysmal atrial fibrillation: the aftherapy study. *Europace* 9 1110–1118. 10.1093/europace/eum253 18042619

[B18] Carlson M. D. Ip J. Messenger J. Beau S. Kalbfleisch S. Gervais P. (2003). A new pacemaker algorithm for the treatment of atrial fibrillation: results of the atrial dynamic overdrive pacing trial (adopt). *J. Am. College Cardiol.* 42 627–633. 10.1016/s0735-1097(03)00780-0 12932592

[B19] Castro-Torres Y. Carmona-Puerta R. Katholi R. E. (2015). Ventricular repolarization markers for predicting malignant arrhythmias in clinical practice. *World J. Clin. Cases* 3:705. 10.12998/wjcc.v3.i8.705 26301231 PMC4539410

[B20] Chen T. Guestrin C. (2016). “Xgboost: a scalable tree boosting system,” in *Proceedings of the 22nd Acm Sigkdd International Conference on Knowledge Discovery and Data Mining*, (New York, NY: Association for Computing Machinery), 785–794.

[B21] Christini D. J. Stein K. M. Markowitz S. M. Mittal S. Slotwiner D. Lerman B. (1999). The role of nonlinear dynamics in cardiac arrhythmia control. *Heart Dis.* 1 190–200. 11720623

[B22] Christini D. J. Stein K. M. Markowitz S. M. Mittal S. Slotwiner D. J. Scheiner M. A. (2001). Nonlinear-dynamical arrhythmia control in humans. *Proc. Natl. Acad. Sci. U.S.A.* 98 5827–5832. 10.1073/pnas.091553398 11320216 PMC33298

[B23] Chung M. K. Eckhardt L. L. Chen L. Y. Ahmed H. M. Gopinathannair R. Joglar J. A. (2020). Lifestyle and risk factor modification for reduction of atrial fibrillation: a scientific statement from the american heart association. *Circulation* 141 e750–e772. 32148086 10.1161/CIR.0000000000000748

[B24] Clinic C. (2018). *Arrhythmia.* Treasure Island, FL: StatPearls.

[B25] Clinic C. (2019). *Ambulatory Monitors.* Rochester: Mayo Clinic.

[B26] Clinic M. (2021). *Holter Monitor.* Rochester: Mayo Clinic.

[B27] Connolly S. J. Gent M. Roberts R. S. Dorian P. Roy D. Sheldon R. S. (2000). Canadian implantable defibrillator study (cids) a randomized trial of the implantable cardioverter defibrillator against amiodarone. *Circulation* 101 1297–1302. 10725290 10.1161/01.cir.101.11.1297

[B28] Coplan N. L. Fuster V. (1990). Limitations of the exercise test as a screen for acute cardiac events in asymptomatic patients. *Am. Heart J.* 119 987–990. 10.1016/s0002-8703(05)80352-7 2321524

[B29] Costin H. Rotariu C. Pãsãricã A. (2013). “Atrial fibrillation onset prediction using variability of ecg signals,” in *Proceedings of the 2013 8th International symposium on advanced topics in electrical engineering (ATEE)*, Bucharest, 1–4.

[B30] De Asmundis C. Conte G. Sieira J. Chierchia G.-B. Rodriguez-Manero M. Di Giovanni G. (2014). Comparison of the patient-activated event recording system vs. Traditional 24 h holter electrocardiography in individuals with paroxysmal palpitations or dizziness. *Europace* 16 1231–1235. 10.1093/europace/eut411 24574492

[B31] de Voogt W. van Hemel N. de Vusser P. Mairesse G. H. van Mechelen R. Koistinen J. (2007). No evidence of automatic atrial overdrive pacing efficacy on reduction of paroxysmal atrial fibrillation. *Europace* 9 798–804. 10.1093/europace/eum149 17670782

[B32] Desai D. S. Hajouli S. (2021). *Arrhythmias.* Treasure Island, FL: StatPearls.

[B33] Dimmer C. Szili-Torok T. Tavernier R. Verstraten T. Jordaens L. (2003). Initiating mechanisms of paroxysmal atrial fibrillation. *Europace* 5 1–9. 10.1053/eupc.2002.0273 12504634

[B34] Dubljevic S. Christofides P. D. (2008). Optimal mechano-electric stabilization of cardiac alternans. *Chem. Eng. Sci.* 63 5425–5433.

[B35] Dubois R. Shah A. J. Hocini M. Denis A. Derval N. Cochet H. (2015). Non-invasive cardiac mapping in clinical practice: application to the ablation of cardiac arrhythmias. *J. Electrocardiol.* 48 966–974. 10.1016/j.jelectrocard.2015.08.028 26403066

[B36] Dvir H. Zlochiver S. (2013). Stochastic cardiac pacing increases ventricular electrical stability—a computational study. *Biophys. J.* 105 533–542. 10.1016/j.bpj.2013.06.012 23870274 PMC3714878

[B37] Ferreira B. B. de Paula A. S. Savi M. A. (2011). Chaos control applied to heart rhythm dynamics. *Chaos Solitons Fractals* 44 587–599.

[B38] Florea V. G. Cohn J. N. (2014). The autonomic nervous system and heart failure. *Circulation Res.* 114 1815–1826. 24855204 10.1161/CIRCRESAHA.114.302589

[B39] Florek J. B. Girzadas D. (2018). *Amiodarone.* Treasure Island, FL: StatPearls.

[B40] García-Civera R. Ruiz-Granell R. Morell-Cabedo S. Sanjuan-Mañez R. Ferrero A. Martínez-Brotons A. (2005). Significance of tilt table testing in patients with suspected arrhythmic syncope and negative electrophysiologic study. *J. Cardiovasc. Electrophysiol.* 16 938–942. 10.1111/j.1540-8167.2005.50029.x 16174011

[B41] Garfinkel A. Spano M. L. Ditto W. L. Weiss J. N. (1992). Controlling cardiac chaos. *Science* 257 1230–1235. 10.1126/science.1519060 1519060

[B42] Garfinkel A. Weiss J. N. Ditto W. L. Spano M. L. (1995). Chaos control of cardiac arrhythmias. *Trends Cardiovasc. Med.* 5 76–80. 10.1016/1050-1738(94)00083-2 21232241

[B43] Germano J. J. Reynolds M. Essebag V. Josephson M. E. (2006). Frequency and causes of implantable cardioverter-defibrillator therapies: is device therapy proarrhythmic? *Am. J. Cardiol.* 97 1255–1261. 10.1016/j.amjcard.2005.11.048 16616037

[B44] Gold M. R. Van Veldhuisen D. J. Hauptman P. J. Borggrefe M. Kubo S. H. Lieberman R. A. (2016). Vagus nerve stimulation for the treatment of heart failure: the inovate-hf trial. *J. Am. Coll. Cardiol.* 68 149–158. 27058909 10.1016/j.jacc.2016.03.525

[B45] Goldberger J. J. Arora R. Buckley U. Shivkumar K. (2019). Autonomic nervous system dysfunction: Jacc focus seminar. *J. Am. College Cardiol.* 73 1189–1206. 10.1016/j.jacc.2018.12.064 30871703 PMC6958998

[B46] Goldsmith S. R. (2004). Interactions between the sympathetic nervous system and the raas in heart failure. *Curr. Heart Fail. Rep.* 1 45–50. 16036024 10.1007/s11897-004-0024-5

[B47] Golińska A. K. Lesiński W. Przybylski A. Rudnicki W. R. (2020). Towards prediction of heart arrhythmia onset using machine learning. *Int. Conf. Comput. Sci.* 12140 376–389. 10.1016/j.cmpb.2018.07.014 30337081

[B48] Grandi E. Ripplinger C. M. (2019). Antiarrhythmic mechanisms of beta blocker therapy. *Pharmacol. Res.* 146:104274. 10.1016/j.phrs.2019.104274 31100336 PMC6679787

[B49] Gürdal A. Eroğlu H. Helvaci F. Sümerkan M. Ç Kasali K. Çetin Ş (2017). Evaluation of tp-e interval, tp-e/qt ratio and tp-e/qtc ratio in patients with subclinical hypothyroidism. *Ther. Adv. Endocrinol. Metab.* 8 25–32. 28377800 10.1177/2042018816684423 PMC5363453

[B50] Hall M. Todd D. (2006). Modern management of arrhythmias. *Postgraduate Med. J.* 82 117–125. 10.1136/pgmj.2005.033654 16461474 PMC2596702

[B51] Haverkamp W. Hindricks G. Gülker H. (1990). Antiarrhythmic properties of beta-blockers. *J. Cardiovasc. Pharmacol.* 16 S29–S32. 11527133

[B52] Hazim A. Belhamadia Y. Dubljevic S. (2015). Control of cardiac alternans in an electromechanical model of cardiac tissue. *Comput. Biol. Med.* 63 108–117. 10.1016/j.compbiomed.2015.05.011 26069933

[B53] Heo J. Yoon J. G. Park H. Kim Y. D. Nam H. S. Heo J. H. (2019). Machine learning–based model for prediction of outcomes in acute stroke. *Stroke* 50 1263–1265. 10.1161/strokeaha.118.024293 30890116

[B54] Hermosillo A. Falcon J. Marquez M. Arteaga D. Cardenas M. (1999). Positive head-up tilt table test in patients with the long qt syndrome. *Europace* 1 213–217. 10.1053/eupc.1999.0056 11220556

[B55] Hilfiker G. Schoenenberger A. W. Erne P. Kobza R. (2015). Utility of electrophysiological studies to predict arrhythmic events. *World J. Cardiol.* 7:344. 10.4330/wjc.v7.i6.344 26131339 PMC4478569

[B56] Hill N. R. Sandler B. Mokgokong R. Lister S. Ward T. Boyce R. (2020). Cost-effectiveness of targeted screening for the identification of patients with atrial fibrillation: evaluation of a machine learning risk prediction algorithm. *J. Med. Econ.* 23 386–393. 10.1080/13696998.2019.1706543 31855091

[B57] Hindricks G. Potpara T. Dagres N. Arbelo E. Bax J. J. Blomström-Lundqvist C. (2021). 2020 ESC guidelines for the diagnosis and management of atrial fibrillation developed in collaboration with the european association for cardio-thoracic surgery (eacts) the task force for the diagnosis and management of atrial fibrillation of the european society of cardiology (esc) developed with the special contribution of the european heart rhythm association (ehra) of the esc. *Eur. Heart J.* 42 373–498. 32860505 10.1093/eurheartj/ehaa612

[B58] Hoffmann E. Janko S. Steinbeck G. Edvardsson N. Camm J. (2000). Analysis of onset mechanisms of atrial fibrillation in pacemaker patients. *Heart* 83 656–669.

[B59] Hohnloser S. H. Healey J. S. Gold M. R. Israel C. W. Yang S. van Gelder I. (2012). Atrial overdrive pacing to prevent atrial fibrillation: insights from assert. *Heart Rhythm* 9 1667–1673. 10.1016/j.hrthm.2012.06.012 22698765

[B60] Huikuri H. V. Mäkikallio T. H. Perkiömäki J. (2003). Measurement of heart rate variability by methods based on nonlinear dynamics. *J. Electrocardiol.* 36 95–99. 14716599 10.1016/j.jelectrocard.2003.09.021

[B61] Jackman W. M. Zipes D. P. (1982). Low-energy synchronous cardioversion of ventricular tachycardia using a catheter electrode in a canine model of subacute myocardial infarction. *Circulation* 66 187–195. 10.1161/01.cir.66.1.187 7083506

[B62] Jackson J. E. (2005). *A User’s Guide to Principal Components.* Hoboken, NJ: John Wiley & Sons.

[B63] Jamil-Copley S. Bokan R. Kojodjojo P. Qureshi N. Koa-Wing M. Hayat S. (2014). Noninvasive electrocardiographic mapping to guide ablation of outflow tract ventricular arrhythmias. *Heart Rhythm.* 11 587–594. 10.1016/j.hrthm.2014.01.013 24440381 PMC4067940

[B64] Jelinek M. V. Lown B. (1974). Exercise stress testing for exposure of cardiac arrhythmia. *Prog. Cardiovasc. Dis.* 16 497–522. 4590953 10.1016/0033-0620(74)90008-5

[B65] Jeong D. U. Taye G. T. Hwang H.-J. Lim K. M. (2021). Optimal length of heart rate variability data and forecasting time for ventricular fibrillation prediction using machine learning. *Comput. Math. Methods Med.* 2021:6663996. 10.1155/2021/6663996 PMC10435312 37601811

[B66] Jin Z. Sun Y. Cheng A. C. (2009). “Predicting cardiovascular disease from real-time electrocardiographic monitoring: an adaptive machine learning approach on a cell phone,” in *Proceedings of the 2009 Annual International Conference of the IEEE Engineering in Medicine and Biology Society*, (Piscataway, NJ: IEEE), 6889–6892. 10.1109/IEMBS.2009.5333610 19964449

[B67] John R. M. Tedrow U. B. Koplan B. A. Albert C. M. Epstein L. M. Sweeney M. O. (2012). Ventricular arrhythmias and sudden cardiac death. *Lancet* 380 1520–1529. 23101719 10.1016/S0140-6736(12)61413-5

[B68] Johnson R. A. Hutter J. R. A. M. Desanctis R. W. Yurchak P. M. Leinbach R. C. Harthorne J. W. (1974). Chronic overdrive pacing in the control of refractory ventricular arrhythmias. *Ann. Intern. Med.* 80 380–383. 10.7326/0003-4819-80-3-380 4205841

[B69] Jordan P. N. Christini D. J. (2004). Adaptive diastolic interval control of cardiac action potential duration alternans. *J. Cardiovasc. Electrophysiol.* 15 1177–1185. 10.1046/j.1540-8167.2004.04098.x 15485444

[B70] Kakade V. Zhao X. Tolkacheva E. G. (2013). Using dominant eigenvalue analysis to predict formation of alternans in the heart. *Phys. Rev. E* 88:e052716. 10.1103/PhysRevE.88.052716 24329305

[B71] Khurshid S. Choi S. H. Weng L.-C. Wang E. Y. Trinquart L. Benjamin E. J. (2018). Frequency of cardiac rhythm abnormalities in a half million adults. *Circulation* 11:e006273. 10.1161/CIRCEP.118.006273 29954742 PMC6051725

[B72] Kneller J. (2015). Ventricular overdrive pacing during SVT: an opportunity for EP techs and allied professionals. *EP Lab Digest* 15.

[B73] Kowal R. C. Wasmund S. L. Smith M. L. Sharma N. Carayannopoulos G. N. Le B. (2004). Biventricular pacing reduces the induction of monomorphic ventricular tachycardia: a potential mechanism for arrhythmia suppression. *Heart Rhythm.* 1 295–300. 10.1016/j.hrthm.2004.05.008 15851173

[B74] Krittanawong C. Virk H. U. H. Bangalore S. Wang Z. Johnson K. W. Pinotti R. (2020). Machine learning prediction in cardiovascular diseases: a meta-analysis. *Sci. Rep.* 10 1–11. 32994452 10.1038/s41598-020-72685-1 PMC7525515

[B75] Krogh-Madsen T. Christini D. J. (2012). Nonlinear dynamics in cardiology. *Annu. Rev. Biomed. Eng.* 14 179–203. 22524390 10.1146/annurev-bioeng-071811-150106 PMC3733460

[B76] Kulkarni K. Lee S. W. Kluck R. Tolkacheva E. G. (2018a). Real-time closed loop diastolic interval control prevents cardiac alternans in isolated whole rabbit hearts. *Ann. Biomed. Eng.* 46 555–566. 10.1007/s10439-018-1981-2 29356998 PMC5862753

[B77] Kulkarni K. Xie X. Fernandez de Velasco E. M. Anderson A. Martemyanov K. A. Wickman K. (2018b). The influences of the m2r-girk4-rgs6 dependent parasympathetic pathway on electrophysiological properties of the mouse heart. *PLoS One* 13:e0193798. 10.1371/journal.pone.0193798 29668674 PMC5905881

[B78] Kulkarni K. Lee S. W. Tolkacheva E. G. (2016). “Pro-arrhythmic effect of heart rate variability during periodic pacing,” in *Proceedings of the 2016 38th Annual International Conference of the IEEE Engineering in Medicine and Biology Society (EMBC)*, Orlando, FL, 149–152. 10.1109/EMBC.2016.7590662 28268301

[B79] Kulkarni K. Merchant F. M. Kassab M. B. Sana F. Moazzami K. Sayadi O. (2019). Cardiac alternans: mechanisms and clinical utility in arrhythmia prevention. *J. Am. Heart Assoc.* 8:e013750. 31617437 10.1161/JAHA.119.013750 PMC6898836

[B80] Kulkarni K. Sevakula R. K. Kassab M. B. Nichols J. Roberts J. D. Isselbacher E. M. (2021a). Ambulatory monitoring promises equitable personalized healthcare delivery in underrepresented patients. *Eur. Heart J. Digital Health* 2 494–510. 10.1093/ehjdh/ztab047 34604759 PMC8482046

[B81] Kulkarni K. Singh J. P. Parks K. A. Katritsis D. G. Stavrakis S. Armoundas A. A. (2021b). Low-level tragus stimulation modulates atrial alternans and fibrillation burden in patients with paroxysmal atrial fibrillation. *J. Am. Heart Assoc.* 10:e020865. 10.1161/JAHA.120.020865 34075778 PMC8477868

[B82] Kulkarni K. Stavrakis S. Elkholey K. Singh J. P. Parks K. A. Armoundas A. A. (2021c). Microvolt t-wave alternans is modulated by acute low-level tragus stimulation in patients with ischemic cardiomyopathy and heart failure. *Front. Physiol.* 12:707724. 10.3389/fphys.2021.707724 34366894 PMC8343129

[B83] Kulkarni K. Walton R. D. Armoundas A. A. Tolkacheva E. G. (2021d). Clinical potential of beat-to-beat diastolic interval control in preventing cardiac arrhythmias. *J. Am. Heart Assoc.* 10:e020750. 34027678 10.1161/JAHA.121.020750 PMC8483541

[B84] Kulkarni K. Tolkacheva E. G. (2021). Nonlinear analytical approaches for prediction of alternans mediated cardiac arrhythmias. *Highlights Med. Med. Sci.* 5 35–47. 10.9734/bpi/hmms/v5/8498d

[B85] Kulkarni K. Visweswaran R. Zhao X. Tolkacheva E. G. (2015). Characterizing spatial dynamics of bifurcation to alternans in isolated whole rabbit hearts based on alternate pacing. *Biomed. Res. Int.* 2015:170768. 10.1155/2015/170768 26581885 PMC4637012

[B86] Kulkarni N. Mody P. Levine B. D. (2020). Abolish the tilt table test for the workup of syncope! *Circulation* 141 335–337. 10.1161/circulationaha.119.043259 32011926

[B87] Lai D. Zhang Y. Zhang X. Su Y. Heyat M. B. B. (2019). An automated strategy for early risk identification of sudden cardiac death by using machine learning approach on measurable arrhythmic risk markers. *IEEE Access.* 7 94701–94716. 10.1109/access.2019.2925847

[B88] Lantz R. Agarwal A. (2021). A review of amiodarone side effects in a cohort of patients: a quality improvement project. *Chest* 160:A1414. 10.1161/CIRCOUTCOMES.116.003122 27553597 PMC5459602

[B89] Lee D. S. Green L. D. Liu P. P. Dorian P. Newman D. M. Grant F. C. (2003). Effectiveness of implantable defibrillators for preventing arrhythmic events and death: a meta-analysis. *J. Am. College Cardiol.* 41 1573–1582. 10.1016/s0735-1097(03)00253-5 12742300

[B90] Lee G. Sanders P. Kalman J. M. (2012). Catheter ablation of atrial arrhythmias: state of the art. *Lancet* 380 1509–1519. 10.1016/s0140-6736(12)61463-9 23101718

[B91] Lee S. W. Kulkarni K. Annoni E. M. Libbus I. KenKnight B. H. Tolkacheva E. G. (2018). Stochastic vagus nerve stimulation affects acute heart rate dynamics in rats. *PLoS One* 13:e0194910. 10.1371/journal.pone.0194910 29590213 PMC5874066

[B92] Li M. Otani N. F. (2003). Ion channel basis for alternans and memory in cardiac myocytes. *Ann. Biomed. Eng.* 31 1213–1230. 10.1114/1.1616930 14649495

[B93] Lj W. Cy H. Mx L. Hy L. (2010). Arrhythmia after a positive head-up tilt table test. *Zhonghua Xin Xue Guan Bing Za Zhi* 38 805–808. 21092649

[B94] Maury P. Sacher F. Gourraud J.-B. Pasquié J.-L. Raczka F. Bongard V. (2015). Increased tpeak-tend interval is highly and independently related to arrhythmic events in brugada syndrome. *Heart Rhythm.* 12 2469–2476. 10.1016/j.hrthm.2015.07.029 26209263

[B95] McIntyre S. D. Kakade V. Mori Y. Tolkacheva E. G. (2014). Heart rate variability and alternans formation in the heart: the role of feedback in cardiac dynamics. *J. Theor. Biol.* 350 90–97. 10.1016/j.jtbi.2014.02.015 24576615

[B96] Merchant F. M. Sayadi O. Sohn K. Weiss E. H. Puppala D. Doddamani R. (2020). Real-time closed-loop suppression of repolarization alternans reduces arrhythmia susceptibility *in vivo*. *Circ. Arrhythm. Electrophysiol.* 13:e008186. 10.1161/CIRCEP.119.008186 32434448 PMC7334752

[B97] Mirowski M. (1985). The automatic implantable cardioverter-defibrillator: an overview. *J. Am. College Cardiol.* 6 461–466. 10.1016/s0735-1097(85)80186-8 3894475

[B98] Mitchell A. R. Sulke N. (2004). How do atrial pacing algorithms prevent atrial arrhythmias? *EP Europace* 6 351–362. 10.1016/j.eupc.2004.03.005 15172660

[B99] Mohebbi M. Ghassemian H. (2012). Prediction of paroxysmal atrial fibrillation based on non-linear analysis and spectrum and bispectrum features of the heart rate variability signal. *Comput. Methods Prog. Biomed.* 105 40–49. 10.1016/j.cmpb.2010.07.011 20732724

[B100] Moreno A. Walton R. D. Bernus O. Vigmond E. J. Bayer J. D. (2021). Low-energy single-pulse surface stimulation defibrillates large mammalian ventricles. *Heart Rhythm.* [Epub ahead of print]. 10.1016/j.hrthm.2021.10.006 34648972

[B101] Nakagawa H. Beckman K. J. McClelland J. H. Wang X. Arruda M. Santoro I. (1993). Radiofrequency catheter ablation of idiopathic left ventricular tachycardia guided by a purkinje potential. *Circulation* 88 2607–2617. 10.1161/01.cir.88.6.2607 8252671

[B102] Namarvar H. H. Shahidi A. V. (2004). “Cardiac arrhythmias predictive detection methods with wavelet-svd analysis and support vector machines,” in *Proceedings of the The 26th Annual International Conference of the IEEE Engineering in Medicine and Biology Society*, Lyon, 365–368. 10.1109/IEMBS.2004.1403168 17271686

[B103] O’donnell D. Cox D. Bourke J. Mitchell L. Furniss S. (2003). Clinical and electrophysiological differences between patients with arrhythmogenic right ventricular dysplasia and right ventricular outflow tract tachycardia. *Eur. Heart J.* 24 801–810. 10.1016/s0195-668x(02)00654-1 12727147

[B104] Ong M. E. H. Ng C. H. L. Goh K. Liu N. Koh Z. X. Shahidah N. (2012). Prediction of cardiac arrest in critically ill patients presenting to the emergency department using a machine learning score incorporating heart rate variability compared with the modified early warning score. *Crit. Care* 16 1–12. 10.1186/cc11396 22715923 PMC3580666

[B105] Otani N. F. Li M. Gilmour R. F. (2005). What can nonlinear dynamics teach us about the development of ventricular tachycardia/ventricular fibrillation? *Heart Rhythm.* 2 1261–1263. 10.1016/j.hrthm.2005.07.015 16253919

[B106] Papadopoulos C. H. Oikonomidis D. Lazaris E. Nihoyannopoulos P. (2018). Echocardiography and cardiac arrhythmias. *Hellenic J. Cardiol.* 59 140–149. 29203161 10.1016/j.hjc.2017.11.017

[B107] Patel B. Sengupta P. (2020). Machine learning for predicting cardiac events: what does the future hold? *Expert Rev. Cardiovasc. Ther.* 18 77–84. 10.1080/14779072.2020.1732208 32066289 PMC7962010

[B108] Perla H. T. Patloori S. C. S. Manickavasagam A. Chase D. Roshan J. (2021). Do the predictors of right ventricular pacing-induced cardiomyopathy add up?. *Indian Heart J.* 73 582–587. 10.1016/j.ihj.2021.07.011 34627573 PMC8514412

[B109] Petersen P. (1990). Thromboembolic complications in atrial fibrillation. *Stroke* 21 4–13. 10.1161/01.str.21.1.4 2405547

[B110] Petrie A. Zhao X. (2012). Estimating eigenvalues of dynamical systems from time series with applications to predicting cardiac alternans. *Proc. R. Soc. A Math. Phys. Eng. Sci.* 468 3649–3666. 10.1371/journal.pcbi.1002399 22396631 PMC3291525

[B111] Poole-Wilson P. A. Swedberg K. Cleland J. G. Di Lenarda A. Hanrath P. Komajda M. (2003). Comparison of carvedilol and metoprolol on clinical outcomes in patients with chronic heart failure in the carvedilol or metoprolol european trial (comet): randomised controlled trial. *Lancet* 362 7–13. 12853193 10.1016/S0140-6736(03)13800-7

[B112] Prabhu M. A. Pillai V. Shenthar J. (2017). Comparison of efficacy, pattern of response, occurrence of arrhythmias, and the tolerability of nitroglycerine and isoprenaline as provocative drugs during head-up tilt test. *Heart Lung Circ.* 26 586–592. 10.1016/j.hlc.2016.10.006 27988280

[B113] Premchand R. K. Sharma K. Mittal S. Monteiro R. Dixit S. Libbus I. (2014). Autonomic regulation therapy via left or right cervical vagus nerve stimulation in patients with chronic heart failure: results of the anthem-hf trial. *J. Card. Fail.* 20 808–816. 10.1016/j.cardfail.2014.08.009 25187002

[B114] Prudat Y. Madhvani R. V. Angelini M. Borgstom N. P. Garfinkel A. Karagueuzian H. S. (2016). Stochastic pacing reveals the propensity to cardiac action potential alternans and uncovers its underlying dynamics. *J. Physiol.* 594 2537–2553. 10.1113/JP271573 26563830 PMC4850193

[B115] Qin D. Singh J. P. (2020). Low-level tragus stimulation for atrial fibrillation: a glimpse of hope for neuromodulation? *JACC Clin. Electrophysiol.* 6 292–294. 10.1016/j.jacep.2020.01.003 32192679

[B116] Raghunath S. Pfeifer J. M. Ulloa-Cerna A. E. Nemani A. Carbonati T. Jing L. (2021). Deep neural networks can predict new-onset atrial fibrillation from the 12-lead ecg and help identify those at risk of atrial fibrillation–related stroke. *Circulation* 143 1287–1298. 10.1161/CIRCULATIONAHA.120.047829 33588584 PMC7996054

[B117] Ravens U. (2010). Novel pharmacological approaches for antiarrhythmic therapy. *Naunyn Schmiedebergs Arch. Pharmacol.* 381 187–193. 20082192 10.1007/s00210-009-0487-8

[B118] Shakibfar S. Krause O. Lund-Andersen C. Aranda A. Moll J. Andersen T. O. (2019). Predicting electrical storms by remote monitoring of implantable cardioverter-defibrillator patients using machine learning. *Ep Europace* 21 268–274. 10.1093/europace/euy257 30508072

[B119] Singh B. N. (2005). B -adrenergic blockers as antiarrhythmic and antifibrillatory compounds: an overview. *J. Cardiovasc. Pharmacol. Ther.* 10 S3–S14. 10.1177/10742484050100i402 15965570

[B120] Skanes A. C. Krahn A. D. Yee R. Klein G. J. Connolly S. J. Kerr C. R. (2001). Progression to chronic atrial fibrillation after pacing: the canadian trial of physiologic pacing. *J. Am. Coll. Cardiol.* 38 167–172. 10.1016/s0735-1097(01)01326-2 11451268

[B121] Sridhar S. Le D.-M. Mi Y.-C. Sinha S. Lai P.-Y. Chan C. (2013). Suppression of cardiac alternans by alternating-period-feedback stimulations. *Phys. Rev. E* 87:e042712. 10.1103/PhysRevE.87.042712 23679454

[B122] Srinivasan N. T. Schilling R. J. (2018). Sudden cardiac death and arrhythmias. *Arrhythm Electrophysiol. Rev.* 7 111–117. 29967683 10.15420/aer.2018:15:2 PMC6020177

[B123] Stavrakis S. Kulkarni K. Singh J. P. Katritsis D. G. Armoundas A. A. (2020a). Autonomic modulation of cardiac arrhythmias: methods to assess treatment and outcomes. *JACC Clin. Electrophysiol.* 6 467–483. 32439031 10.1016/j.jacep.2020.02.014 PMC7370838

[B124] Stavrakis S. Stoner J. A. Humphrey M. B. Morris L. Filiberti A. Reynolds J. C. (2020b). Treat af (transcutaneous electrical vagus nerve stimulation to suppress atrial fibrillation): a randomized clinical trial. *JACC Clin. Electrophysiol.* 6 282–291. 10.1016/j.jacep.2019.11.008 32192678 PMC7100921

[B125] Stouffer G. A. Sheahan R. G. Lenihan D. J. (2001). Syncope and arrhythmias: role of the electrophysiological study. *Am. J. Med. Sci.* 322 37–43. 11465245 10.1097/00000441-200107000-00007

[B126] Strik M. Ramirez F. D. Welte N. Bonnin T. Abu-Alrub S. Eschalier R. (2020). Progressive implantable cardioverter-defibrillator therapies for ventricular tachycardia: the efficacy and safety of multiple bursts, ramps, and low-energy shocks. *Heart Rhythm.* 17 2072–2077. 10.1016/j.hrthm.2020.07.032 32739474

[B127] Strogatz S. (1994). *Nonlinear Dynamics and Chaos.* Boulder, CO: Avalon Publishing.

[B128] Sulke N. Silberbauer J. Boodhoo L. Freemantle N. Kamalvand K. O’Nunain S. (2007). The use of atrial overdrive and ventricular rate stabilization pacing algorithms for the prevention and treatment of paroxysmal atrial fibrillation: the pacemaker atrial fibrillation suppression (pafs) study. *Europace* 9 790–797. 10.1093/europace/eum111 17562750

[B129] Taye G. T. Shim E. B. Hwang H.-J. Lim K. M. (2019). Machine learning approach to predict ventricular fibrillation based on qrs complex shape. *Front. Physiol.* 10:1193. 10.3389/fphys.2019.01193 31616311 PMC6764170

[B130] Teodorovich N. Swissa M. (2016). Tilt table test today-state of the art. *World J. Cardiol.* 8:277. 10.4330/wjc.v8.i3.277 27022459 PMC4807316

[B131] Tiwari P. Colborn K. L. Smith D. E. Xing F. Ghosh D. Rosenberg M. A. (2020). Assessment of a machine learning model applied to harmonized electronic health record data for the prediction of incident atrial fibrillation. *JAMA Netw. Open* 3:e1919396. 10.1001/jamanetworkopen.2019.19396 31951272 PMC6991266

[B132] Trohman R. G. Huang H. D. Larsen T. Krishnan K. Sharma P. S. (2020). Sensors for rate-adaptive pacing: how they work, strengths, and limitations. *J. Cardiovasc. Electrophysiol.* 31 3009–3027. 10.1111/jce.14733 32877004

[B133] Trohman R. G. Kim M. H. Pinski S. L. (2004). Cardiac pacing: the state of the art. *Lancet* 364 1701–1719. 10.1016/s0140-6736(04)17358-3 15530632

[B134] Tse G. Gong M. Wong W. T. Georgopoulos S. Letsas K. P. Vassiliou V. S. (2017). The tpeak- tend interval as an electrocardiographic risk marker of arrhythmic and mortality outcomes: a systematic review and meta-analysis. *Heart Rhythm.* 14 1131–1137. 28552749 10.1016/j.hrthm.2017.05.031

[B135] Tse G. Yan B. P. (2017). Traditional and novel electrocardiographic conduction and repolarization markers of sudden cardiac death. *Ep Europace* 19 712–721. 10.1093/europace/euw280 27702850

[B136] Vassilikos V. Dakos G. Chatzizisis Y. S. Chouvarda I. Karvounis C. Maynard C. (2011). Novel non-invasive p wave analysis for the prediction of paroxysmal atrial fibrillation recurrences in patients without structural heart disease: a prospective pilot study. *Int. J. Cardiol.* 153 165–172. 10.1016/j.ijcard.2010.08.029 20837368

[B137] Vijayaraman P. Bordachar P. Ellenbogen K. A. (2017). The continued search for physiological pacing: where are we now? *J. Am. Coll. Cardiol.* 69 3099–3114. 10.1016/j.jacc.2017.05.005 28641799

[B138] Wellens H. Smeets J. (1993). Idiopathic left ventricular tachycardia. Cure by radiofrequency ablation. *Circulation* 88 2978–2979. 10.1161/01.cir.88.6.2978 8252711

[B139] Wilson D. Ermentrout B. (2017). Stochastic pacing inhibits spatially discordant cardiac alternans. *Biophys. J.* 113 2552–2572. 10.1016/j.bpj.2017.10.001 29212008 PMC5768519

[B140] Yang A. Hochhäusler M. Schrickel J. Bielik H. Shlevkov N. Schimpf R. (2003). Advanced pacemaker diagnostic features in the characterization of atrial fibrillation: impact on preventive pacing algorithms. *Pacing Clin. Electrophysiol.* 26 310–313. 10.1046/j.1460-9592.2003.00039.x 12687835

[B141] Zagrodzky J. D. Ramaswamy K. Page R. L. Joglar J. A. Sheehan C. J. Smith M. L. (2001). Biventricular pacing decreases the inducibility of ventricular tachycardia in patients with ischemic cardiomyopathy. *Am. J. Cardiol.* 87 1208–1210. 10.1016/s0002-9149(01)01498-9 11356402

[B142] Zannad F. De Ferrari G. M. Tuinenburg A. E. Wright D. Brugada J. Butter C. (2015). Chronic vagal stimulation for the treatment of low ejection fraction heart failure: results of the neural cardiac therapy for heart failure (nectar-hf) randomized controlled trial. *Eur. Heart J.* 36 425–433. 10.1093/eurheartj/ehu345 25176942 PMC4328197

[B143] Zehir R. Karabay C. Y. Kalaycı A. Akgün T. Kılıçgedik A. Kırma C. (2015). Evaluation of tpe interval and tpe/qt ratio in patients with slow coronary flow. *Anatolian J. Cardiol.* 15:463. 10.5152/akd.2014.5503 25430412 PMC5779137

